# Nujiangexathone A, a Novel Compound Derived from *Garcinia nujiangensis*, Induces Caspase-Dependent Apoptosis in Cervical Cancer through the ROS/JNK Pathway

**DOI:** 10.3390/molecules21101360

**Published:** 2016-10-12

**Authors:** Li Zhang, Si-Yuan Kong, Zhao-Qing Zheng, Xiao-Xiao Meng, Ji-Ling Feng, Hong-Sheng Tan, Yuan-Zhi Lao, Lian-Bo Xiao, Hong-Xi Xu

**Affiliations:** 1 School of Pharmacy, Shanghai University of Traditional Chinese Medicine, No. 1200, Cailun Road, Shanghai 201203, China; zhangli1003ecpu@126.com (L.Z.); mo2jiaojiaozhu@163.com (S.-Y.K.); bishopjiaozhu@gmail.com (Z.-Q.Z.); diablo2_joe@163.com (X.-X.M.); fjlfreda@hotmail.com (J.-L.F.); ths97029@163.com (H.-S.T.); laurence.ylao@gmail.com (Y.-Z.L.); 2 Institute of Arthritis Research, Shanghai Academy of Chinese Medical Sciences, Guanghua Integrative Medicine Hospital, No. 540, Xinhua Road, Shanghai 201203, China

**Keywords:** Nujiangexathone A, ROS, JNK, apoptosis, cervical cancer

## Abstract

Nujiangexathone A (NJXA), a novel compound derived from *Garcinia nujiangensis*, has been demonstrated to inhibit the proliferation of several human cancer cell lines. This study is the first to demonstrate the apoptosis inductive activities of NJXA and the possible underlying mechanisms. Our results demonstrated that NJXA inhibited colony formation by HeLa and SiHa cells in a dose-dependent manner. An Annexin V-FITC/PI staining assay showed that NJXA strongly triggered apoptosis in a dose-dependent manner. Western blotting analyses showed that NJXA induced the caspase-dependent apoptosis of HeLa and SiHa cells by triggering a series of events, including changes in the levels of Bcl-2 family proteins, cytochrome *c* release, caspase-3 activation, and chromosome fragmentation. Furthermore, we demonstrated that NJXA induced cell apoptosis by activating the reactive oxygen species (ROS)-mediated JNK signaling pathway. Consistent with this finding, a ROS scavenger, *N*-acetyl-l-cysteine (NAC, 10 mM), hindered NJXA-induced apoptosis and attenuated the sensitivity of HeLa and SiHa cells to NJXA. In vivo results further confirmed that the tumor inhibitory effect of NJXA was partially through the induction of apoptosis. Taken together, our results demonstrated that NJXA induced the apoptosis of HeLa and SiHa cells through the ROS/JNK signaling pathway, indicating that NJXA could be important candidate for the clinical treatment of cervical cancer.

## 1. Introduction

Cancer is a deadly disease that kills approximately half a million people annually. Cervical cancer is the fourth most common type of cancer in women, and approximately 80% of cervical cancers occur in developing countries [1]. Currently, the standard treatment for cervical cancer is radical pelvic surgery followed by chemotherapy. Patients are generally treated with cisplatin-based chemotherapy; however, adverse side effects, including nephrotoxicity, and gastrointestinal and cardiovascular damage, have limited the success of this treatment. To date, all cytotoxic anticancer drugs have various harmful side effects when used alone or in combination, and their lack of selectivity for cancer cells continues to be the greatest challenge for anticancer drug discovery [2]. Therefore, there is a high demand for effective and selective pharmaceutical agents to treat cervical cancer.

Natural products derived from herbal medicines have tremendous potential as promising drug candidates; for decades, medicinal herbs have been a major source of the drugs that have been discovered [3]. *Garcinia* species (from the Guttiferae family) are tropical evergreen trees and shrubs that are widely distributed in Southeastern Asia. This genus consists of 450 species, of which 21 species are found throughout China [4]. *Garcinia* species contain many special compounds, including xanthones, benzophenones, bioflavonoids, and biphenyls. Garcinia extract is generally used as a traditional Chinese medicine for detoxification and the treatment of inflammation and wounds [5,6]. In the last decade, most of the research on *Garcinia* species has focused on their anticancer activity. Gambogic acid, a caged xanthone derived from *Garcinia hanburyi*, has been tested in vitro and in vivo as a novel anticancer agent that inhibits cell proliferation, angiogenesis, and metastasis [7,8,9]. *Garcinia nujiangensis* is a Chinese endemic species that is found mainly in the southwestern region of China. Nujiangexanthone A (NJXA), a novel compound isolated from the leaves of *Garcinia nujiangensis*, exhibited cytotoxicity against a panel of human tumor cell lines. However, the mechanisms underlying the anticancer activity of NJXA remain elusive.

Currently, one of the major goals of cancer drug development is inducing tumor cell apoptosis [10,11,12]. Caspases play important roles in the activation and execution of apoptosis [13]. Some caspases, such as caspase-8 and -9, are ‘‘initiators’’ of the apoptotic process; whereas others, such as caspase-3, -6, and -7, are ‘‘executioners’’ of this process. Poly(ADP-ribose) polymerase (PARP) is the substrate of caspase-3, and PARP cleavage causes the disassembly of cell structures and DNA fragmentation, which eventually leads to cell death [14,15]. Other key regulators of apoptosis are members of the Bcl-2 protein family. Based on their functions, these proteins can be divided into pro-apoptotic and anti-apoptotic classes. The pro-apoptotic Bcl-2 proteins, such as Bax and Bak, activate apoptosis by permeabilizing the mitochondrial outer membrane and destroying mitochondrial integrity, which causes cell death [16,17]. In contrast, the anti-apoptotic Bcl-2 proteins, including Bcl-2 and Bcl-xL, maintain mitochondrial integrity, thus preventing apoptotic cell death [18,19].

Reactive oxygen species (ROS) are chemically reactive oxygen-containing compounds that are formed as a natural byproduct of normal metabolism. ROS have important roles in cell signaling and homeostasis, with a low level of ROS improving cellular function and cell survival and a high level of ROS, leading to the oxidative damage of lipids, proteins, and DNA via their effect on various cellular signaling pathways, including the MAPK signal transduction pathway [20,21]. JNK is a member of the MAPK family and is responsive to stress stimuli, which play vital roles in cell proliferation, motility, metabolism, DNA repair, and death [22]. The role of the ROS/JNK signaling pathway in cancer cell apoptosis has been widely studied, and this pathway is an important target of cancer drug therapies [11,23,24].

In this study, we evaluated the in vitro and in vivo anticancer effects of NJXA and elucidated the molecular mechanism underlying these effects. The results indicated that NJXA induced apoptosis through the ROS-mediated JNK signaling pathway and inhibited tumor growth in a HeLa xenograft model. Taken together, we provide solid evidence that NJXA is a potential candidate for the future treatment of cervical cancer.

## 2. Results

### 2.1. NJXA Inhibits the Proliferation and Induced the Death of Cervical Cancer Cells

**Figure 1:**
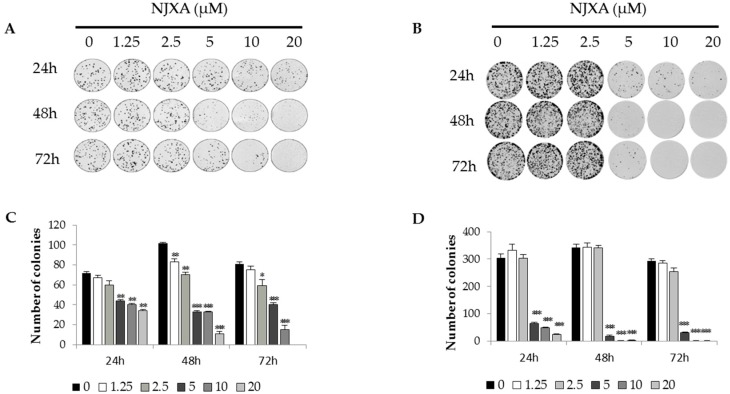
NJXA inhibits the proliferation of cervical cancer cells. (**A**) HeLa and (**B**) SiHa cells were seeded in 6-well plates at 200 cells per well. After 24 h of growth, the cells were treated with NJXA (0~20 μM) for 24, 48, and 72 h. Then, the media were replaced with complete medium without drugs, and the cells were cultured for 14 days. The colony counts for (**C**) HeLa and (**D**) SiHa cells are presented. The data shown are the mean values ± SD. * *p* < 0.05, ** *p* < 0.01, *** *p* < 0.001 compared with the control. *n* = 3.

A previous investigation showed that NJXA exhibited toxicity toward several cancer cell lines [25]. To study the potential effect of NJXA on long-term proliferation, we used a colony formation assay. HeLa or SiHa cells were seeded at 200 cells per well and were treated with 0~20 μM of NJXA for 24, 48, and 72 h. The results indicated that NJXA suppressed the formation of colonies by both cervical cancer cell lines in a concentration- and time-dependent manner (Figure 1). After treatment with 20 μM of NJXA for 72 h, very few colonies were found in the HeLa cell cultures. NJXA had a stronger inhibitory effect on SiHa cells than on HeLa cells, with their colony formation almost completely inhibited after treatment with 10 μM of NJXA for 48 h. These results demonstrated that NJXA exhibited highly selective cytotoxicity toward the cervical cancer cells tested without exerting obvious cytotoxicity toward normal cells [26].

### 2.2. NJXA Induces Caspase-Dependent Apoptosis in HeLa and SiHa Cells

**Figure 2:**
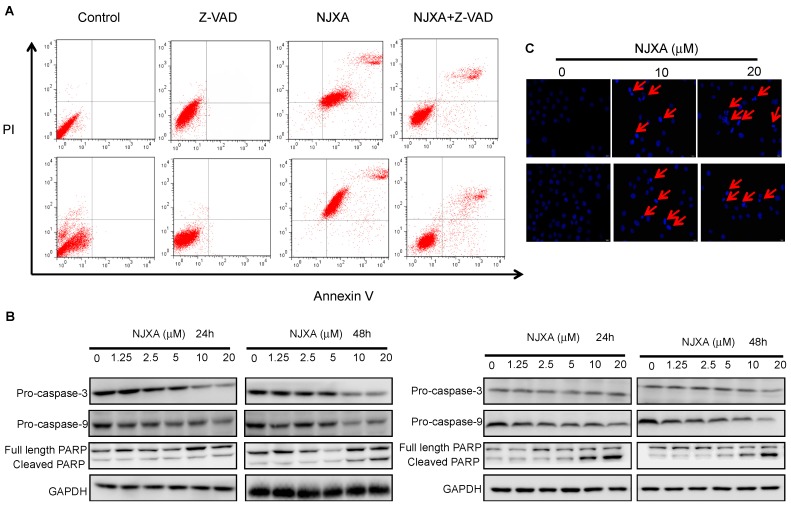
NJXA triggers apoptosis in HeLa and SiHa cells. (**A**) Annexin V/PI flow cytometric analysis of NJXA-treated HeLa (upper panel) and SiHa (lower panel) cells. Cells pre-treated with z-VAD-fmk for 2 h were then treated with or without NJXA (20 μM) for 24 h. The cells were then collected and were double-stained with a FITC-conjugated anti-Annexin V antibody and PI. The analyses were performed using a flow cytometer; (**B**) Western blotting analysis showed caspase-3 and caspase-9 activation and PARP cleavage in HeLa (**left panel**) and SiHa (**right panel**) cells treated with NJXA; (**C**) Hoechst 33342 staining showed DNA condensation and fragmentation after NJXA treatment of HeLa (**upper panel**) and SiHa (**lower panel**) cells.

To investigate the effect of NJXA on HeLa and SiHa cells, we performed an apoptosis assay in which flow cytometric analysis of human HeLa and SiHa cells treated with 20 μM of NJXA for 24 h and then double-stained with propidium iodide (PI), and an anti-Annexin V antibody was conducted. As shown in Figure 2A, the number of apoptotic cells in both the HeLa and SiHa cell populations was significantly increased by NJXA treatment. To confirm these findings, we investigated the involvement of caspases in the effect of NJXA using the caspase inhibitor z-VAD-fmk. As expected, the Annexin V/PI flow cytometric apoptosis assay showed that the apoptosis of HeLa and SiHa cells treated with NJXA (20 μM) for 24 h after a 2-h pre-treatment with z-VAD-fmk was strongly inhibited (Figure 2A). We also found that there was a fraction of cells near the border of the top right quadrant that seems insensitive to z-VAD, which were possibly the necrotic cells, where damaged plasma membrane permits penetration of Annexin V and binding PS in the internal membrane layer.

Additionally, the apoptosis of NJXA-treated cells was confirmed by Western blotting analysis of the activities of caspase-dependent pathway markers, including caspase-3, caspase-9, and PARP. Compared with their levels in the control cells, the activities of caspase-3 and caspase-9 were elevated in the cells treated for 24 and 48 h with NJXA because they contained decreased amounts of pro-caspase-3 and pro-caspase-9, whereas the amount of cleaved PARP was significantly increased in the treated cells (Figure 2B). Hoechst 33342 staining also showed that NJXA induced the development of the morphological characteristics of apoptosis. DNA condensation and fragmentation were initially observed after treatment with 10 μM of NJXA for 48 h and significantly increased when the concentration of NJXA was increased to 20 μM (Figure 2C).

### 2.3. NJXA Activates the Mitochondria-Dependent Apoptotic Pathway in Cervical Cancer Cells

It has been suggested that the Bax-mediated mitochondrial signaling pathway plays an important role in apoptosis [16,17]. In our study, the key events following the activation of the mitochondrial signaling pathway, including changes in the levels of Bcl-2 family proteins, cytochrome *c* release, mitochondrial fission, and swelling, were examined in cells undergoing NJXA-induced apoptosis.

**Figure 3:**
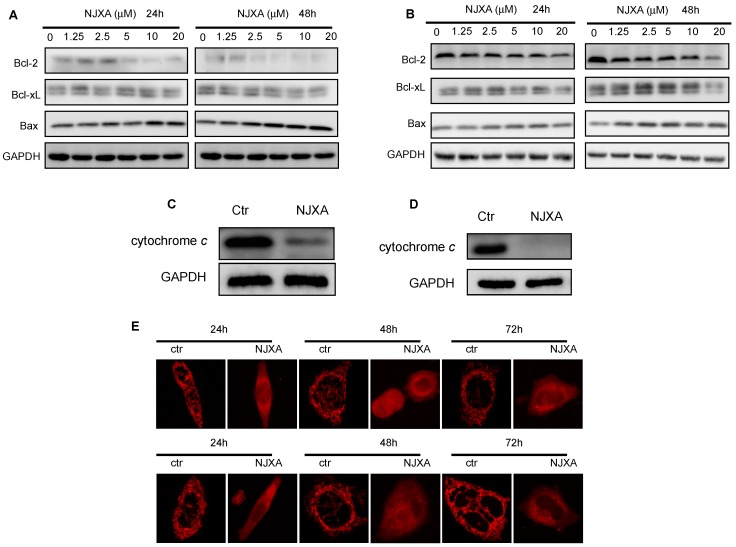
NJXA induces mitochondria-dependent apoptosis in HeLa and SiHa cells. (**A**) HeLa or (**B**) SiHa cells were treated with NJXA (0~20 μM) for 24 h or 48 h, and then Bcl-2, Bcl-xL, and Bax levels were analyzed by Western blotting; (**C**) NJXA induced cytochrome *c* release in HeLa and (**D**) SiHa cells. The mitochondrial and cytosolic fractions of cells treated with NJXA (20 μM) for 72 h were analyzed by Western blotting for cytochrome *c* and GAPDH; (**E**) MitoTracker Red staining showed that NJXA induced mitochondrial fission and swelling in HeLa (**upper panel**) and SiHa (**lower panel**) cells.

The Western blotting results showed that the levels of the anti-apoptotic Bcl-2 proteins, including Bcl-2 and Bcl-xL, were decreased in a concentration- and time-dependent manner after NJXA treatment in both HeLa and SiHa cells, whereas the level of the pro-apoptotic protein Bax was increased (Figure 3A,B). We also assessed the release of cytochrome *c* in the treated cells. As shown in Figure 3C,D, NJXA dramatically reduced the amount of cytochrome *c* in the mitochondria of the cervical cancer cells. These results indicated that NJXA induces Bax-mediated mitochondrial cytochrome *c* release. We also examined the changes in mitochondrial morphology induced by NJXA treatment by staining cells with a fluorescent dye, MitoTracker Red. As shown in Figure 3E, in normal HeLa and SiHa cells stained with MitoTracker Red, the mitochondria have filamentous morphology. However, upon 20-μM of NJXA treatment, the mitochondria underwent fission and swelling, which may have been due to the loss of the mitochondrial membrane potential.

### 2.4. NJXA Regulates Cervical Cancer Cell Apoptosis via Activating the JNK Pathway

**Figure 4:**
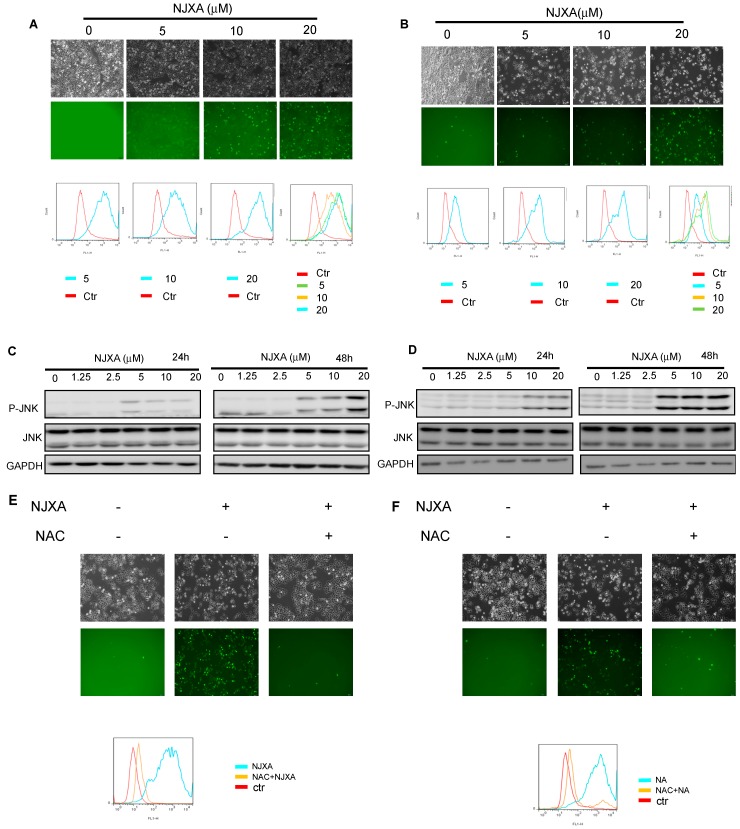

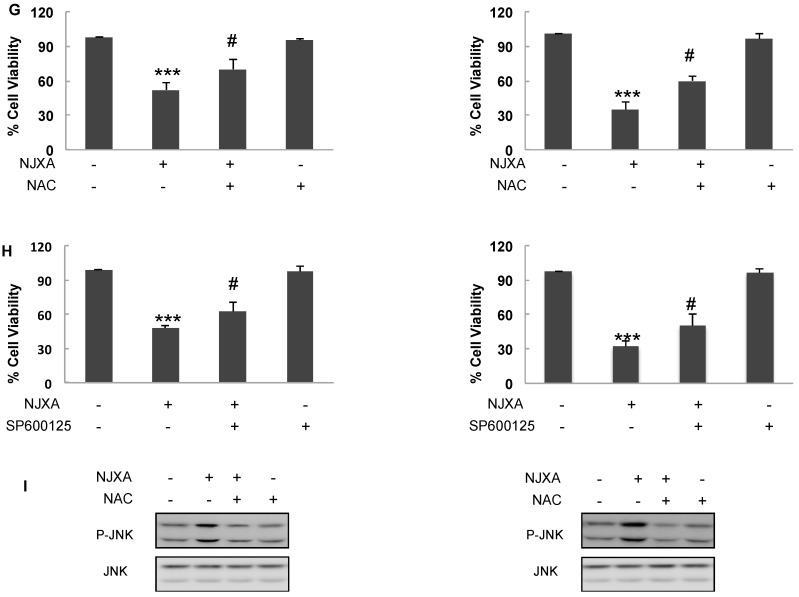
NJXA activates the ROS/JNK pathway in HeLa and SiHa cells. (**A**) HeLa or (**B**) SiHa cells were treated with NJXA (0~20 μM) for 12 h. The cells were incubated with DCFH-DA and were observed under a fluorescence microscope (**upper panel**) or were collected and were analyzed using flow cytometry (**lower panel**); (**C**) HeLa or (**D**) SiHa cells were treated with NJXA (0~20 μM) for 24 h or 48 h, and their p-JNK levels were then analyzed by Western blotting; (**E**) HeLa or (**F**) SiHa cells were pretreated with 10 mM of NAC for 2 h and then were incubated with DMSO or 20 μM of NJXA for 12 h. The cells were treated with DCFH-DA and were observed under a fluorescence microscope (**upper panel**) or were collected and were analyzed using flow cytometry (**lower panel**); (**G**) HeLa (**left panel**) or SiHa (**right panel**) cells were treated with NJXA (20 μM) for 48 h after pretreatment with NAC (10 mM) for 2 h. Cell viability was measured by MTT assay; (**H**) HeLa (**left panel**) or SiHa (**right panel**) cells were treated with NJXA for 48 h after pretreatment with SP600125 (20 μM) for 2 h. Cell viability was (measured by MTT assay. The data shown are the mean values ± SD. *** *p* < 0.001 compared with the control, ^#^ *p <* 0.05 compared (with NJXA. *n* = 3; (**I**) Western blotting analysis for protein expression of phospho-JNK and JNK was measured in HeLa (**left panel**) or SiHa (**right panel**) treated with NJXA after pretreatment with NAC (10 mM) for 2 h.

ROS are known to participate in regulating apoptosis [27] and to promote the activation of JNK by inhibiting MAP kinase phosphatases [28]. In our study, the level of intracellular ROS was measured using DCFH-DA. Fluorescence microscopy revealed that cells exposed to NJXA for 12 h had much brighter green fluorescence than did the cells in the control group (Figure 4A,B), indicating that NJXA induced the marked accumulation of intracellular ROS. Flow cytometric analysis also demonstrated that the level of intracellular ROS increased in a dose-dependent manner in the treated cells when the concentration of NJXA was increased from 0 μM to 20 μM. Because JNK is a stress response protein kinase that can be activated by ROS [29], we investigated the effect of NJXA on JNK in HeLa and SiHa cells. Western blotting analysis showed that the level of total JNK was not obviously changed after NJXA treatment compared with the level in the control cells, whereas NJXA treatment triggered the concentration-dependent upregulation of the level of phosphorylated JNK (Figure 4C,D). Moreover, pretreatment with the antioxidant NAC at 10 mM counteracted the increase in intracellular ROS that occurred in HeLa and SiHa cells exposed to NJXA (Figure 4E,F), as shown by fluorescence microscopic and flow cytometric analysis. The proportion of apoptotic cells was also decreased obviously in the presence of NAC or SP600125 in NJXA-treated cells (Figure 4G,H). At the same time, NAC effectively antagonized the phosphorylation of JNK in response to NJXA (Figure 4I). Therefore, these results indicate that ROS contributed to the NJXA-induced apoptosis of HeLa and SiHa cells.

### 2.5. NJXA Has a Significant Antitumor Effect against Xenografted Tumors in A Nude Mice Model

**Figure 5:**
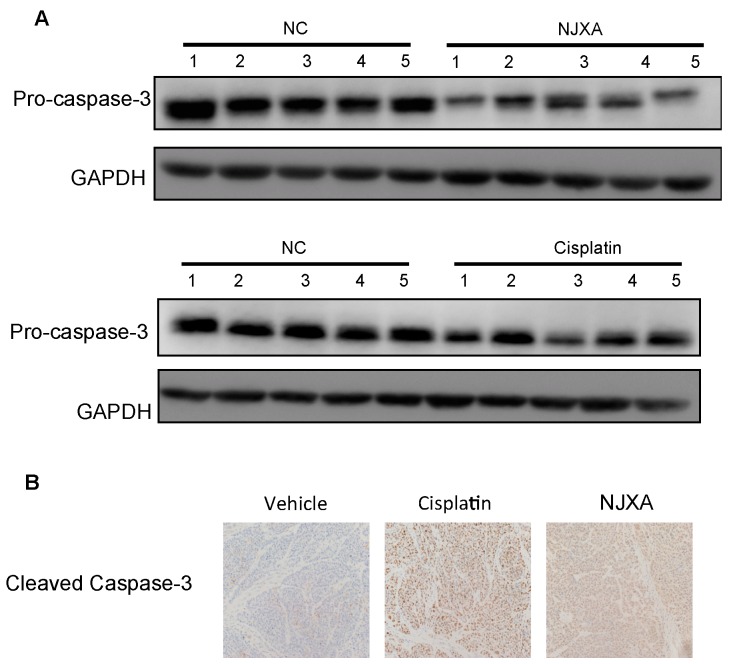
NJXA induced cleavage of pro-caspase-3 in xenografted tumors. HeLa cells (1 × 10^6^ cells per mouse) were injected subcutaneously into the dorsal flank of nude mice. When the tumors reached approximately 75 mm^3^, the mice received a daily intraperitoneal injection of either the vehicle control (1% Tween-80 in normal saline) or NJXA (20 mg/kg) or an injection of cisplatin (2 mg/kg) every 2 days. After treatment for 16 days, the mice were sacrificed. (**A**) Western blotting analysis of the expression levels of pro-caspase-3 and GAPDH protein in mouse tumor sections; (**B**) Representative images of tumor sections immunohistochemically stained to examine the cleaved caspase-3 level.

The antitumor effect of NJXA was examined in nude mice, as described previously [26]. Our previous results showed that NJXA treatment decreased both the volume and weight of the xenografted tumors compared with those in the control group and that the anticancer activity of NJXA was comparable to that of cisplatin [26]. To better understand the molecular mechanism underlying the effect of NJXA on tumorigenesis in vivo, we examined the expression level of pro-caspase-3 and the level of cleaved caspase-3 in tumor tissues using Western blotting and immunohistochemical analysis, respectively. As shown in Figure 5A, the expression levels of pro-caspase-3 were significantly lower in the tumor tissues of NJXA-treated mice than in those of the control mice, whereas the level of cleaved caspase-3 was dramatically increased. These results strongly indicated that NJXA inhibited the growth of cervical cancer in vivo by inducing apoptosis.

## 3. Discussion

In this study, the in vitro and in vivo antitumor activity and mechanism underlying this activity of NJXA, a novel compound isolated from *G*. *nujiangensis*, was investigated with regard to its potential development as an anticancer drug for treating cervical cancer. Apoptosis is a programmed cell death process that occurs in multicellular organisms, which can be activated through two different pathways. One of these activating pathways is an intrinsic pathway that is generally initiated by stimulation with chemical compounds or irradiation. The other such pathway is an extrinsic pathway that is activated by the binding of death ligands to the death receptors [19]. It is believed that apoptosis has a close association with many diseases, with deficient apoptosis causing the loss of controlled proliferation, which eventually results in carcinogenesis [30]. Insights into antitumor activities have given rise to optimism regarding the development of cancer-cell-specific therapies [10,12]. It has been observed that many plant-derived chemotherapeutic drugs kill cancer cells by promoting apoptotic cell death [31,32,33,34]. In this study, we demonstrated that NJXA triggers the activation of the mitochondria-dependent apoptotic signaling pathway, resulting in the increase in pro-apoptotic protein levels, cytochrome *c* release, caspase activation, PARP cleavage, DNA fragmentation, and apoptosis in HeLa and SiHa cells. These results indicated that NJXA was toxic and lethal to HeLa and SiHa cells through inducing apoptosis.

The ROS-JNK pathway is involved in NJXA-induced apoptosis. A low dosage of ROS improves cellular function and enhances cell survival. However, excessive ROS promotes programmed cell death [35]. The results of the present study indicated that the ROS level in human cervical cancer cells treated with NJXA was significantly increased. The ROS inhibitor NAC was found to prevent these changes, suggesting that ROS are involved in the NJXA-induced apoptosis. Studies have demonstrated that the excessive generation of ROS imbalances cellular homeostasis via affecting multiple signaling pathways [20] and that JNK played significant roles in many of the resulting cellular events [29]. Our present study showed that treating HeLa and SiHa cells with NJXA resulted in the upregulation of the JNK1/2 levels, as shown using Western blotting. Based on these data, we concluded that JNK is involved in the NJXA-induced apoptosis of cervical cancer cells and that the mechanism underlying the NJXA-mediated antitumor effect was associated with the ROS-JNK interaction. Our present results are also consistent with the results of previous studies showing that ROS-induced JNK activation was involved in the apoptosis of some types of tumor cells [35,36].

The results of in vivo study indicated that NJXA could inhibit the development of xenografted tumors partially through the induction of apoptosis. Our previous study indicated that NJXA also showed tumor inhibitory effects via the down-regulation of heterogeneous nuclear ribonucleoprotein K (hnRNPK) by accelerating ubiquitin-proteasome-dependent hnRNPK degradation, which then induced cell cycle arrest through the c-Myc-cyclin/Cdk-Rb-E2F1 pathway. Unlike the Western drug, which usually has a single target, the compounds from natural plants are often multi-targets. As we reported previously that the compound oblongifolin C (OC), a PPAP purified from *G. yunnanensis* Hu, showed tumor inhibitory effects via different mechanisms: (i) activation of mitochondria-dependent apoptotic pathway [19]; (ii) inhibition of autophagic flux [37]; (iii) inhibition of metastasis via upregulation of keratin 18 and tubulins [38]; and induction of DNA damage and ER stress [39]. Our study here indicated that both apoptosis and cell circle arrest contribute to the anti-tumor effect of NJXA. The detailed mechanisms of NJXA still need to be investigated further.

## 4. Materials and Methods

### 4.1. Cell Lines and Cell Culture

Human cervical cancer carcinoma HeLa and SiHa cell lines were cultured in Dulbecco’s modified Eagle’s medium (Gibco/Invitrogen, 12800-017, Carlsbad, CA, USA) supplemented with 10% fetal bovine serum (PAA, A15-101) and 10 U/mL of penicillin–streptomycin (Gibco/Invitrogen, 15140-122) at 37 °C in a humidified 5% CO_2_ incubator.

### 4.2. Annexin V-FITC/PI Staining Assay

After treatment, the cells were harvested, washed twice with ice-cold PBS, and then stained using an Annexin V-FITC/PI Cell Apoptosis Detection Kit (Thermo Fisher Scientific, Waltham, MA, USA) according to the manufacturer's instructions. In brief, 1 × 10^6^ cells were resuspended in 400 μL of binding buffer and then 5 μL of 2 mg/mL of an Annexin V antibody, and 5 μL of 20 μg/mL PI were added. After 15 min of incubation in the dark, apoptosis was quantified using BD FACS Calibur flow cytometer (Becton & Dickinson Company, Franklin Lakes, NJ, USA), and the data were analyzed with the FlowJo 7.6.1 software (FlowJo, LLC, Ashland, OR, USA). The cells in the early stage of apoptosis were Annexin V positive and PI negative, whereas the cells in the late stage of apoptosis were both Annexin V and PI positive.

### 4.3. Western Blotting Analysis

Cell lysates containing 20 μg of protein were fractionated using SDS-PAGE, and then the proteins were transferred to a polyvinylidene difluoride membrane. After blocking nonspecific binding with TBS/T (0.1%) containing 5% non-fat milk for 1 h at room temperature, the membrane was incubated with different antibodies. The membrane was washed four times with TBS/T to remove the unbound antibodies and then was incubated with the HRP-conjugated secondary antibodies at room temperature for 1 h. The labeled protein bands were visualized using an ECL kit (Pierce, Rockford, IL, USA).

### 4.4. Colony Formation Assay

Two hundred cells per well were seeded in 6-well plates, and the plates were incubated overnight. The cells were then treated with NJXA at various concentrations for the indicated periods. After being rinsed with fresh medium, the cells were allowed to form colonies for 14 days and were subsequently fixed and stained with 0.04% crystal violet. Colonies observed under a microscope to contain more than 50 cells were counted as one positive colony.

### 4.5. Hoechst Staining Assay

After treatment, the cells were incubated with Hoechst 33342 (10 μg/mL) for 5 min at room temperature in the dark. Then, the stained cells were observed under a fluorescence microscope.

### 4.6. ROS Production Assay

Intracellular ROS production was measured as described previously [40,41]. In brief, cells were stained with DCFH-DA for 30 min at 37 °C in the dark. The stained cells were rinsed with PBS and then were observed immediately under a fluorescence microscope (Olympus IX 83, Tokyo, Japan). The absorbance at 488 nm (excitation wavelength)/525 nm (emission wavelength) was measured.

To determine the intensity of fluorescence, cells were stained with DCFH-DA for 30 min at 37 °C in the dark. After 3 rinses with phosphate-buffered saline (PBS), the cells were digested using 0.25% trypsin and were re-suspended in PBS, and the intensity of fluorescence was measured by flow cytometry according to the manufacturer’s instructions.

### 4.7. Cell Viability Assay

Cells were pretreated with 10 mM of NAC (Sigma, Taufkirchen, Germany,) or 20 μM of SP600125 (Cell Signaling Technology, Danvers, MA, USA) for 2 h, and then treated with NJXA (20 μM) for 24 h. After treatment, a 3-(4,5-dimethylthiazol-2-yl)2,5-diphenyltetrazolium bromide (MTT) solution was added and incubated for 4 h. After the medium was removed, 100 μL of DMSO was added, the absorbance was measured at 570 nm, and cell viability was normalized as the percentage of control.

### 4.8. Subcellular Fractionation

After treatment, cells were harvested by trypsin-EDTA treatment, and the cytosolic and mitochondrial fractions were separated using a Cell Mitochondria Isolation Kit (Beyotime, Shanghai, China, C3601) according to the manufacturer's instructions.

### 4.9. In Vivo Animal Study

Four-week old BALB/c nude mice were purchased from the Experimental Animal Center of the Chinese Academy of Science (Shanghai, China) and were maintained in a pathogen-free environment in the Experimental Animal Center of the Shanghai University of Traditional Chinese Medicine. The experimental procedures were approved by the Shanghai University of Traditional Chinese Medicine Committee for the Use of Live Animals for Teaching and Research and were conducted in accordance with the Guide for the Care and Use of Laboratory Animals, published by the NIH [publication No. SCXX (HU) 2007-0005]. HeLa cells (1 × 10^6^ cells per mouse) were subcutaneously inoculated into the dorsal flank of nude mice. When the tumor dimensions reached ~75 mm^3^, the mice were randomly divided into the three following groups (*n* = 6 per group): (1) vehicle (1% Tween-80 in saline); (2) NJXA, 20.0 mg/kg every day; and (3) cisplatin, 2 mg/kg every other day. At the end of the experimental periods (16 days after tumor implantation), the mice were sacrificed, and their tumors were removed. A portion of each tumor was fixed in 10% paraformaldehyde (PFA) for the immunohistochemical assay, and the rest was used for Western blotting analysis.

### 4.10. Hematoxylin Staining and Immunohistochemistry

Tumor samples were fixed in 10% PFA at 4 °C for 48 h. Selected samples were embedded in paraffin, sectioned, and stained with a cleaved caspase-3 (CST, 9662) antibody. The primary antibody was used at a dilution of 1:100. Finally, the sections were mounted with DPX Mountant (Sigma, 317616) for histological analysis.

### 4.11. Statistical Analyses

The data are presented as the mean values ± SD from three independent experiments. The statistical analysis was performed using the Student 2-tailed *t-*test. *p*-values of less than 0.05 were considered to indicate significant differences.

## 5. Conclusions

Our study demonstrated the mechanism through which NJXA induced apoptosis in cervical cancer cells. The NJXA-mediated induction of apoptosis in cervical cancer cells was regulated through the ROS-mediated JNK signaling pathway. These data, combined with the evidence obtained using an animal model, strongly support a role for NJXA as a novel anticancer drug candidate for the therapy of cervical cancer through the induction of apoptosis.
